# COVID-19 and mental health of older Africans: an urgency for public health policy and response strategy

**DOI:** 10.1017/S1041610220003312

**Published:** 2020-08-24

**Authors:** Razak M Gyasi

**Affiliations:** Aging and Development Unit, African Population and Health Research Center (APHRC), Nairobi, Kenya

## Introduction

The novel coronavirus disease 2019 (COVID-19) has emerged as a global public health challenge and profoundly continues to inundate governments, economies, health systems, and social lives. Although COVID-19 has swept persons of any age cohort in all regions and territories globally, the extent of the reverberations remains almost uncertain. What we know, however, is that persons aged 60 years or older are highly exposed to the pandemic and constitute a disproportionate chunk of the fatalities (Mahase, 2020; Verity *et al*., 2020; WHO, 2020a), engendering ardent fear and “moral panic” among older populations.

The age factor has been linked to the life course and age-related declines in immune functioning and the likelihood of having severe preexisting chronic comorbidities, such as cardiovascular disease (CVD) and pulmonary conditions (Gyasi and Anderson, 2020; Nikolich-Zugich *et al*., 2020), which unfortunately predominate in older age (Gyasi and Phillips, 2020). This development has serious implications for the COVID-19 policy and public health response. However, like the 2014–2016 Ebola outbreak in West Africa, the respective national strategies to control the COVID-19 contagion and clinical response in sub-Saharan Africa (SSA) have not been tailored to the specific care needs of the highly at-risk older populations. The current trend of the infection in Africa warrants good policy and sensible public health planning.

While approximately 70% of global older people reside in low- and middle-income countries (LMICs), health systems in these settings have weak capacity and also lack methodic epidemiological surveillance systems to contain the potential surge of the infection and the concomitant debilitating impacts (Gilbert *et al.*, 2020). Many SSA countries have limited public health infrastructure, personnel, and other resources such as intensive care unit beds, ventilators, isolation centers, and personal protective equipment (PPEs) to fight COVID-19 (WHO, 2020b). Looming is the potential straining of the health systems with the spread of the virus and the growing number of critically ill older patients (Grasselli *et al.*, 2020). Obviously, SSA’s struggle to fight the threat of COVID-19 may be a *fiasco* if no state-of-the-art countermeasures are procured. Our efforts could be seriously undermined given the rise of chronic comorbidities and cognitive disabilities in the region particularly among older adults (Gyasi and Phillips, 2020) as well as the heaves of societal ills, such as fragile economies, chronic poverty, and income and food insecurities.

Unfortunately, there is a paucity of gerontological and geriatric expertise and services to enhance social and emotional well-being healing of the COVID-19 infected and affected older people in most African countries (Gyasi, 2020a). Interrogating the mental health impacts of COVID-19 for older age in SSA is, therefore, a key element of preparedness and effective response effort to the COVID-19 crisis and beyond.

## Aging and mental health in SSA

Trends in demographic aging have been dramatic in SSA. The region is aging at a much faster rate than was the case for today’s richer countries with implications for family structure and worse public mental health outcomes (WHO, 2015). SSA’s older populations are expected to increase from 46 million in 2015 to 161 million by 2050, representing approximately 11.3% of the total population of the region (WHO, 2018). Many older adults will feel empty nest, extremely lonely, and socially isolated with limited intergenerational support chiefly as a result of the growing trend of modernization and the current influx of outmigration among the young people in SSA. Demand for long-term and palliative care services will likely upsurge due to age-related disabilities and chronic conditions such as HIV/AIDS, CVD, stroke, diabetes, and cancers. Coupled with poor socioeconomic and environmental conditions, many older persons in Africa are expected to experience serious neuropsychiatric disorders.

Estimates show that more than 20% of older Africans live with mental or neurological disorders (WHO, 2017). Psychological distress and depression which frequently comorbid with anxiety disorder affect many older people with marked spatial, gender, and age dimensions. Depression accounts for approximately 6% of years lived with disability among older people (WHO, 2017). Studies from the SSA region suggest an increased risk of depression in old age (Mirkena *et al*., 2018; Peltzer and Phaswana-Mafuya, 2013).

Furthermore, the number of people with dementia, particularly, among the oldest olds is projected to be around 8 million by 2050, about 250% increase from current numbers (WHO, 2019). Moreover, a population-based study estimates that approximately 90% of older people with dementia and Alzheimer’s disease in Central African Republic experience neuropsychiatric disorders over the course of their disease (Yoro-Zohoun *et al.*, 2019). Other studies have reported similar estimates among Nigerian and Tanzanian older adults (Ojagbemi, Akinyemi, and Baiyewu, 2013; Sumari-de Boer *et al.*, 2017). However, dementias largely remain “hidden” in SSA. Many people either do not report it or fail to seek care due to both widespread systemic (e.g. defective case finding techniques) and social (e.g. traditional beliefs and stigmatization) challenges. Very importantly, the situation is expected to aggravate during the COVID-19 viral pandemic, a time when health care seeking for chronic and mental conditions is vehemently disrupted particularly among the poor.

Indeed, dementias and other mental/cognitive impairments add a great deal to the impacts of non-communicable diseases (NCDs) and disability in SSA and represent a major public health burden on many older people and their families (Gyasi and Phillips, 2020; Vik-Mo *et al.*, 2020). The WHO has also formally recognized dementias such as Alzheimer’s disease as a cause of death in SSA rather than just a disability (WHO, 2015). Nevertheless, mental illnesses and their long-term psychological effects constitute the less readily acknowledged NCDs in SSA. The emergence of the SARS-CoV-2, massive later life fatalities, and political emergency decisions to contain the spread of the virus may worsen mental health risks and the existing psychiatric symptoms among older Africans (Yang *et al.*, 2020).

## The COVID-19 crisis, social isolation and mental health in older Africans

In March 2020, countries in the WHO SSA region reported their index cases of COVID-19 (WHO, 2020a). Although the contagion is relatively recent in SSA, an exponential curve is being manifested. The most recent data (as of 29 August 2020) suggest that 1,029,787 confirmed cases of COVID-19 with 21,151 deaths, a 3.8% fatality rate is recorded in the region (WHO, 2020b; Worldometer, 2020). The case-fatality ratio by age stood at 14.8% for 80+, 8.0% for 70–79, and 3.6% for 60–69 years olds compared to 0.2–0.3% for those under 45 years of age (Lloyd-Sherlock *et al.*, 2020; WHO, 2020a). The severe age-segregated fatalities and fear of COVID-19 infection have created serious emotional insecurity and major depressive and anxiety disorders among older Africans. Unfortunately, many older people are subjected to ageist scapegoating (Gyasi and Phillips, 2020). Others are neglected, experience domestic abuse, and have age-related discrimination. Reports from Ghana indicate that people reject a hospital ward where older adults with other medical problems (rather than COVID-19) are admitted. These elder abuses and discriminatory behaviors toward older persons have the tendency to heighten the mental health challenges of older Africans with dire implications for health and policy response to COVID-19 in SSA.

The global scientific community has directed much attention toward finding treatment for COVID-19. However, the science behind the development of effective pharmacological interventions or vaccine for COVID-19 has not been fully understood (as of August 2020). It, therefore, remains highly erratic about how the SARS-COV-2 will evolve. Based on the WHO guidelines for country-level response to COVID-19, governments in SSA implemented and in certain cases intensified strict quarantine, “spatial” or “social” distancing, self-isolation, and hand hygiene measures to keep the trend of COVID-19 toward the flattening of new cases. However, these policies and decisions have been made under uncertain and poor economic conditions that have constrained access to basic services, such as water and sanitation in the region. Moreover, frail, mentally, and chronically ill older people may be deprived of palliative, long-term, and routine health care services chiefly because the COVID-19 emergencies and responses portend to absorb the scant health resources. These have unleashed a threat to older people who are already disproportionately vulnerable to the pandemic, showing adverse impacts on their mental and psychological health.

It is well known that older people are at a greater risk not only of COVID-19 but also the diverse inevitable sequelae of the pandemic including the separation and isolation from families and caregivers. Like many LMICs, the novel practices of emergency lockdowns in all SSA countries have resulted in changes in national behavioral patterns of usual day-to-day functioning and have escalated poverty and hunger particularly among poor older people (Gyasi, 2020a; Gyasi, 2020b; Sonenthal *et al.*, 2020). The nationwide prohibition on the social gathering by closing down religious activities and stringent directives on the use of public transport as well as the mass quarantine have inevitably become a major barrier to access maintenance treatments for this group. While online mental health services are being adopted and promoted, particularly, in the urban SSA, many older people either have limited access to smartphones and internet services or are not technology savvy. Under these circumstances, older adults with psychiatric disorders may not have access to psychiatric clinics and maintenance medications. Further, the restrictions have stirred a sudden separation of older people from loved ones, caused a shortage of living supplies, intense depression and loneliness, loss of freedom, and psychological insecurity (Gyasi, 2020a).

Intergenerational solidarity and *filial piety* norms in many countries such as Ghana and Kenya have been viciously negated under the stringent lockdown regulations, particularly in areas where migration of the young people is in vogue. The mental health impacts of social isolation among older adults in SSA are related to previous disease outbreaks such as the 2014–2016 Ebola epidemic. In Sierra Leone, Liberia, and Uganda, the deterioration of social relationships was explained by the vulnerability of older people. In *International Psychogeriatrics*, Ayalon (2020) advocates that the ability of older people to survive outbreaks of these kind is further weakened under lockdown regulations. Although these measures are important to contain the community spread of the virus, they will inevitably have consequences on short- and long-term mental health effects and well-being for older people.

## Forfending COVID-19-related mental health impacts in SSA

The concerns of the disparaging impacts of COVID-19 on the mental health of older Africans are gradually catching research and social policy attention. However, fragile health systems and psychogeriatric services and corruption are still highly prevalent in SSA. Juxtaposed with the experiences from the past epidemics, would it be easy to deal head-on with the drastic impacts of COVID-19 in SSA? The “first port of call” is to acknowledge the existence and the extent of the challenge (Lloyd-Sherlock *et al.*, 2020). In our struggle to overcome older age mental health impacts of this pandemic, a priority should focus on key proactive areas of action.

First, social distancing should be understood as “*spatial separation*” but not *social disconnection*. Social distancing does not mean one cannot stay close to their families. Social integration and support have proven to be important psychosocial resources for emotional development in SSA (Gyasi *et al.*, 2019; Wand *et al.*, 2020). Averting COVID-19-related mental problems, families and friends should be recognized as the primary and indispensable frontline care providers. New ways of maintaining frequent social connections and interactions with older adults, that is, the use of digital technologies such as video and audio phone calls/chats will not only bridge “spatial” distance (Merchant and Lurie, 2020) but also as effective psychogeriatric services to reduce anxiety and depression among older people. Workplace and family virtual/video meetings can be vital to maintain social contact and improve emotional health among older people (Galea *et al.*, 2020; Hwang *et al.*, 2020; Vedavanam *et al.*, 2020).

Issues about loneliness as a result of the physical—rather than social—isolation are widespread. Individual older people should be made to understand that loneliness is different from solitude. Being alone does not mean one is lonely (Rokach and Sha’ked, 2013). People could be in a company of loved ones but will still feel quite lonely. Loneliness is largely a perception which could be mitigated through positive imagination and thoughts. Effective education and sensitization from relatives and media can be a critical resource for older people (Gyasi, Phillips and Abass, 2019).

Engagement in regular physical activity such as performing moderate household activities and talking/movement in the compound and outside the home may serve as an important psychosocial and *cultural* therapy for older people. Indoor physical exercise might be a potential therapy to maintain robust physical health and counteract the psychological impact of the pandemic. These are effective interventions for loneliness and depression (Gyasi, 2019; López-Torres Hidalgo *et al.*, 2019) and also as a coping strategy particularly for older adults. Older people should be encouraged to participate in regular exercises to avert and or minimize the potential mental health impacts of COVID-19.

The global epidemic of misinformation is rapidly spreading via social media platforms and other local communication outlets (Zarocostas, 2020). The WHO Director-General, Tedros Adhanom Ghebreyesus, at the Munich Security Conference on 15 February 2020 asserted that “we’re not just fighting an epidemic; we’re fighting an infodemic”. This could pose serious mental health problems particularly for the at-risk populations including older adults in poor settings. The media have a crucial role to play during COVID-19 and that ensuring a responsible mass media will be a useful avenue to manage older Africans’ mental health problems (Buffel *et al.*, 2020). Electronic media should be sanitized and desist from reporting false and inaccurate information about the pandemic. Unprofessionalism of the media and unethical media reporting may potentially heighten fear and depression among older people. The COVID-19-related ageist discussions should be avoided. The media should rather concentrate on educating and communicating social and preventive measures using culturally acceptable modes to manage the misinformation and to mitigate mental health impacts.

Pragmatic measures such as sustainable social and pro-poor interventions should be adopted by African governments to prevent extreme poverty and economic hardships which could aggravate mental health problems during the fight against COVID-19. Vulnerable older people need social protection policies, including cash and funding support. The government of Ghana, for example, has implemented free access to water for all citizens and a reduction in electricity charges for the poor. More than ever, there will be an urgent need to sustain and expand such response packages, especially for older people. Other SSA countries could also learn from these lessons and apply similar strategies. Synergies between the governments, private sector, and civil society/philanthropists will be particularly important to safeguard older people from the contagion and the attendant mental health issues.

Moreover, COVID-19 has led to diverse challenges for mental health services for older people. Limited attention is being paid to this vulnerable population since much effort is directed to responding to COVID-19. The health systems in the respective SSA countries should adjust and prepare for geriatric care and psychological support for older people who are highly at-risk of the pandemic. Stakeholders and health policymakers should collaborate to resolve this barrier in order to provide high-quality, timely psychological services to older people. Also, culturally oriented educational platforms for psychological counseling services for older people in general and those affected with COVID-19 will increase public trust and safeguard the mental health of those vulnerable to the pandemic. In addition, social media can be used to reassure groups to connect and direct older people to reliable resources for mental health support.

## Conclusions

Like any other global region, the threat of COVID-19 has thrust SSA into uncharted territory. Policy interventions to respond to the spread of the pandemic—emergency lockdowns and “spatial”/“social” distancing modalities—can potentially engender severe mental and behavioral health problems for especially vulnerable population groups, including older adults. The impact of COVID-19 on older people living in challenging settings with limited health resources and with weak capacity to endure the impacts of the pandemic is highly concerning. With the propensity of seeing a second wave and potential for flare-ups of the pandemic, a critical evaluation of cost-effective therapies and interventions to respond to the mental health needs of older people requires integrated attention. This is the time to strengthen our mental and psychiatric health services and to prepare for the inevitably precipitated challenges of the pandemic. Shared responsibility is urgently needed to effectively address the huge mental health and well-being impacts of COVID-19 among older people in SSA. Finally, post-COVID-19 health recovery and mental rehabilitation programs for older people should be included in regional and country-specific development policy agendas.
